# ﻿Two novel *Helicosporium* species (Tubeufiaceae, Tubeufiales) from southern China based on morphological and molecular evidence

**DOI:** 10.3897/mycokeys.124.168933

**Published:** 2025-10-23

**Authors:** Song Bai, Fang Wang, Su-Ran Wan, Xiao-Kang Lv, Li-Jun Chen, Rong Wu, Jian Ma

**Affiliations:** 1 Guizhou Industry Polytechnic College, Guiyang 550008, China Guizhou Industry Polytechnic College Guiyang China; 2 School of Food and Pharmaceutical Engineering, Guizhou Institute of Technology, Guiyang 550003, China School of Food and Pharmaceutical Engineering, Guizhou Institute of Technology Guiyang China

**Keywords:** Dothideomycetes, helicosporous fungi, phylogeny, taxonomy, two novel species

## Abstract

During a survey of saprobic fungi, fresh specimens were collected from decaying wood in terrestrial habitats in Guizhou and Hainan provinces, southern China. Two novel species, *Helicosporium qixianlingense* and *H. tongrenense*, are introduced based on phylogenetic analyses of a combined dataset (ITS, LSU, *tef*1-α, and *rpb*2) and morphological evidence. Comprehensive descriptions, illustrations, notes, and phylogenetic analyses supporting the taxonomic placement of these new taxa are provided. These findings are significant for exploring the species diversity of *Helicosporium* in southern China.

## ﻿Introduction

Tubeufiales (Pleosporomycetidae, Dothideomycetes) was established by Boonmee et al. (2014), with Tubeufiaceae designated as the type family based on both morphological characteristics and molecular DNA data. Currently, the order includes three families: Bezerromycetaceae, Tubeufiaceae, and Wiesneriomycetaceae, as determined through molecular DNA data and divergence time estimates (Boonmee et al. 2011, 2014, 2021; Liu et al. 2017; Wijayawardene et al. 2022). In the latest comprehensive revision by Lu et al. (2018b), based on morphological comparison and/or molecular data, 13 new genera, 52 new species, 16 new records, and 43 new combinations were added to Tubeufiales. Recent studies also revised the classification of previously misidentified Tubeufiaceae species and emphasized the importance of considering all morphological differences when identifying helicosporous asexual species (Lu et al. 2017a,b, 2018a, b, 2022a, 2023a, b; Lu and Kang 2020).

Tubeufiaceae, typified by *Tubeufia* (Barr 1979), is the largest family within Tubeufiales (Wijayawardene et al. 2022; Ma et al. 2024b), encompassing 53 accepted genera, namely *Acanthohelicospora*, *Acanthophiobolus*, *Acanthostigma*, *Acanthostigmina*, *Acanthotubeufia*, *Acrogenihelicosporium*, *Aquaphila*, *Berkleasmium*, *Bifrontia*, *Boerlagiomyces*, *Camporesiomyces*, *Chaetosphaerulina*, *Chlamydotubeufia*, *Dematiohelicoma*, *Dematiohelicomyces*, *Dematiohelicosporum*, *Dematiotubeufia*, *Dictyospora*, *Discotubeufia*, *Excipulariopsis*, *Helicangiospora*, *Helicoarctatus*, *Helicodochium*, *Helicohyalinum*, *Helicoma*, *Helicomyces*, *Helicosporium*, *Helicotruncatum*, *Helicotubeufia*, *Hyalohelicoon*, *Hyalohelicotubeufia*, *Hyalohelisphora*, *Kamalomyces*, *Kevinhydea*, *Lichenotubeufia*, *Manoharachariella*, *Muripulchra*, *Neoacanthostigma*, *Neochlamydotubeufia*, *Neohelicoma*, *Neohelicomyces*, *Neohelicosporium*, *Neomanoharachariella*, *Neotubeufia*, *Parahelicomyces*, *Pleurohelicosporium*, *Pseudohelicoon*, *Pseudohelicosporium*, *Pseudotubeufia*, *Tamhinispora*, *Thaxteriella*, *Tubeufia*, and *Zaanenomyces* (Boonmee et al. 2011, 2014; Hyde et al. 2016, 2017, 2024; Luo et al. 2017; Lu et al. 2018b; Tibpromma et al. 2018; Liu et al. 2019; Crous et al. 2021, 2023; Ma et al. 2023a, b, 2024b). Among them, *Camporesiomyces* and *Tubeufia* exhibit diverse asexual conidial morphology (Carris 1989; Matsushima 1993; Zhao et al. 2007; Hyde et al. 2020; Han et al. 2025).

Based on morphological evidence, the genus *Helicosporium* was established by Nees (1817), with *H. vegetum* as the type species. Currently, 30 species are accepted within the genus *Helicosporium*, occurring in freshwater and/or terrestrial habitats across diverse regions, including Austria, Belgium, Britain, British Guiana, Canada, China, Cuba, England, France, Germany, India, Thailand, and the USA (Nees 1817; Linder 1929; Samuels and Muller 1979; Barr 1980; Goos 1989; Morgan-Jones and Goos 1992; Spatafora et al. 2006; Tsui et al. 2006; Zhao et al. 2007; Boonmee et al. 2014, 2021; Lu et al. 2017a, 2018b, 2022a; Dong et al. 2020; Hsieh et al. 2021; Xiao et al. 2023; Ma et al. 2024b; Peng et al. 2025; Sun et al. 2025). Based on molecular DNA data, *Helicosporium* species exhibit similar morphological characteristics in both their sexual and asexual morphs (Boonmee et al. 2014, 2021; Lu et al. 2017a, 2018b, 2022a; Dong et al. 2020; Hsieh et al. 2021; Xiao et al. 2023; Ma et al. 2024b; Peng et al. 2025; Sun et al. 2025). For example, the sexual morph is characterized by solitary, greenish, reddish-yellow to brownish-yellow ascomata; cylindric-clavate, eight-spored bitunicate asci; and hyaline to yellowish-brown, elongate-fusiform ascospores (Boonmee et al. 2014, 2021; Brahmanage et al. 2017; Lu et al. 2022a; Peng et al. 2025; Sun et al. 2025). The asexual morph is characterized by pale yellow to yellow-green colonies, erect, setiferous conidiophores, and helicoid, hyaline to yellow-green conidia (Linder 1929; Zhao et al. 2007; Boonmee et al. 2014, 2021; Lu et al. 2017a, 2018b, 2022a; Dong et al. 2020; Hsieh et al. 2021; Xiao et al. 2023; Ma et al. 2024b).

In this study, four newly obtained fungal isolates, representing two distinct *Helicosporium* taxa within the family Tubeufiaceae (Tubeufiales, Dothideomycetes), were collected from decaying wood in southern China. Based on morphological comparisons, illustrations, and multigene phylogenetic analyses of combined ITS, LSU, *tef*1-α, and *rpb*2 sequence data, two novel species, *Helicosporium qixianlingense* and *H. tongrenense*, are introduced in the present study.

## ﻿Materials and methods

### ﻿Sample collection, examination, and isolation

Decaying wood was collected from Qixianling Hot Spring National Forest Park, Hainan Province, and Jiangkou County, Guizhou Province, in southern China. Samples were transported to the laboratory in plastic bags, accompanied by collection details such as locality, habitat, and date (Rathnayaka et al. 2024). Microscopic features were examined and photographed using a stereomicroscope (SMZ-168, Nikon, Japan) and an ECLIPSE Ni compound microscope (Nikon, Tokyo, Japan) equipped with a Canon 90D digital camera. Measurements were taken with Tarosoft Image Frame Work software, and photo plates were prepared using Adobe Photoshop CC 2019 (Adobe Systems, USA).

Single-spore isolations were conducted on potato dextrose agar (PDA) plates following the protocols of Chomnunti et al. (2014) and Senanayake et al. (2020). Germinated conidia or ascospores were aseptically transferred to fresh PDA plates. Morphological characteristics of fungal mycelia on PDA, including colony color, hyphal shape, and growth dimensions, were recorded. Dried fungal specimens were deposited in the Herbarium of Guizhou Academy of Agricultural Sciences (Herb. GZAAS), Guiyang, China, and pure cultures were preserved in the Guizhou Culture Collection (GZCC), Guiyang, China. MycoBank numbers were obtained following the procedures described at https://www.mycobank.org/.

### ﻿DNA extraction, PCR amplification, and sequencing

Fresh fungal mycelia were scraped from PDA-grown colonies and transferred to 1.5-mL microcentrifuge tubes using sterilized lancets for genomic DNA extraction. DNA was extracted with the Biospin Fungus Genomic DNA Extraction Kit (BioFlux, China). The following primer pairs were used for PCR amplification: ITS5/ITS4 for the internal transcribed spacer region (ITS; White et al. 1990), LR0R/LR5 for the large subunit ribosomal RNA gene (LSU; Vilgalys and Hester 1990), EF1-983F/EF1-2218R for translation elongation factor 1-α (*tef*1-α; Rehner and Buckley 2005), and fRPB2-5F/fRPB2-7cR for RNA polymerase II second largest subunit (*rpb*2; Liu et al. 1999). Each 50-μL PCR reaction contained 2 μL of template DNA, 2 μL of each primer, and 44 μL of 1.1× T3 Super PCR Mix (Qingke Biotech, Chongqing, China). Polymerase chain reaction (PCR) was performed using the cycling conditions described by Ma et al. (2024a). Amplification products were purified and sequenced using the same primers at Beijing Tsingke Biotechnology Co., Ltd.

### ﻿Phylogenetic analyses

The newly obtained sequences were quality-checked and assembled using BioEdit v.7.0.5.3 (Hall 1999) and SeqMan v.7.0.0 (DNASTAR, Madison, WI, USA; Swindell and Plasterer 1997). The sequences used in this study were retrieved from GenBank (Table 1; https://www.ncbi.nlm.nih.gov/). Sequence matrices for each gene were aligned using MAFFT v.7.473 (Katoh et al. 2019; https://mafft.cbrc.jp/alignment/server/). Alignments were trimmed with trimAl v.1.2rev59 (Capella-Gutiérrez et al. 2009) and concatenated using SequenceMatrix v.1.7.8 (Vaidya et al. 2011).

**Table 1.** Taxa used in this study and their GenBank accession numbers.

Taxon	Strain	GenBank Accessions			
		ITS	LSU	tef1-α	rpb2
*Acanthohelicospora aurea*	GZCC 16-0060	KY321323	KY321326	KY792600	MF589911
*Acanthostigma chiangmaiensis*	MFLUCC 10-0125^T^	JN865209	JN865197	KF301560	N/A
*Acanthostigma perpusillum*	UAMH 7237	AY916492	AY856892	N/A	N/A
*Berkleasmium aquaticum*	MFLUCC 17-0049^T^	KY790444	KY790432	KY792608	MF535268
*Berkleasmium fusiforme*	MFLUCC 17-1978^T^	MH558693	MH558820	MH550884	MH551007
*Boerlagiomyces macrospora*	MFLUCC 12-0388	KU144927	KU764712	KU872750	N/A
*Botryosphaeria agaves*	MFLUCC 10-0051	JX646790	JX646807	N/A	N/A
*Botryosphaeria dothidea*	CBS 115476	KF766151	DQ678051	DQ767637	DQ677944
*Camporesiomyces bhatii*	GMBCC 1120^T^	PQ763360	PQ842543	PV388894	PV388888
*Chlamydotubeufia cylindrica*	MFLUCC 16-1130^T^	MH558702	MH558830	MH550893	MH551018
*Chlamydotubeufia huaikangplaensis*	MFLUCC10-0926^T^	JN865210	JN865198	N/A	N/A
*Dematiohelicomyces helicosporus*	MFLUCC 16-0213^T^	KX454169	KX454170	KY117035	MF535258
*Dematiohelicosporum guttulatum*	MFLUCC 17-2011^T^	MH558705	MH558833	MH550896	MH551021
*Dematiotubeufia chiangraiensis*	MFLUCC 10-0115^T^	JN865200	JN865188	KF301551	N/A
*Helicangiospora lignicola*	MFLUCC 11-0378^T^	KF301523	KF301531	KF301552	N/A
*Helicoarctatus aquaticus*	MFLUCC 17-1996^T^	MH558707	MH558835	MH550898	MH551024
*Helicohyalinum aquaticum*	MFLUCC 16-1131^T^	KY873625	KY873620	KY873284	MF535257
*Helicohyalinum infundibulum*	MFLUCC 16-1133^T^	MH558712	MH558840	MH550903	MH551029
*Helicoma guttulatum*	MFLUCC 16-0022^T^	KX454171	KX454172	MF535254	MH551032
*Helicoma hongkongense*	MFLUCC 17-2005	MH558716	MH558843	MH550907	MH551033
*Helicosporium acropleurogenum*	CGMCC 3.25563^T^	PP626574	PP639430	PP596333	PP596460
*Helicosporium aquaticum*	MFLUCC 17-2008^T^	MH558733	MH558859	MH550924	MH551049
*Helicosporium brunneisporum*	CGMCC 3.25542^T^	PP626577	PP639433	PP596336	PP596463
*Helicosporium changjiangense*	GZCC 22-2113^T^	PP626578	PP639434	PP596337	PP596464
*Helicosporium flavisporum*	MFLUCC 17-2020^T^	MH558734	MH558860	MH550925	MH551050
*Helicosporium flavum*	MFLUCC 16-1230^T^	KY873626	KY873621	KY873285	N/A
*Helicosporium hainanense*	GZAAS 22-2006^T^	OP508730	OP508770	OP698081	OP698070
*Helicosporium jiangkouense*	HKAS 128933^T^	PP626580	PP639436	PP596339	PP596466
*Helicosporium latisporum*	HKAS 128960^T^	PP626582	PP639437	PP596340	PP596467
*Helicosporium liuzhouense*	GZCC 22-2014^T^	OQ981394	OQ981402	OQ980476	OQ980474
*Helicosporium luteosporum*	MFLUCC 16-0226^T^	KY321324	KY321327	KY792601	MH551056
*Helicosporium multidentatum*	GZCC 22-2013^T^	OQ981395	OQ981403	OQ980477	OQ980475
*Helicosporium multiseptatum*	GUCC 24-0090^T^	PQ570843	PQ570860	PQ761135	N/A
*Helicosporium nanningense*	GZCC 22-2175^T^	OR066418	OR066425	OR058864	OR058857
***Helicosporium qixianlingense***	**GZCC 25-0641^T^**	**PX111181**	**PX111188**	**PX102605**	**PX102599**
***Helicosporium qixianlingense***	**GZCC 25-0642**	**PX111182**	**PX111189**	**PX102606**	**PX102600**
*Helicosporium ramosiphorum*	CGMCC 3.25541^T^	PP626576	PP639432	PP596335	PP596462
*Helicosporium rubrum*	MFLUCC 24-0090^T^	PQ098477	PQ098514	PQ490681	PQ490675
*Helicosporium setiferum*	MFLUCC 17-1994^T^	MH558735	MH558861	MH550926	MH551051
*Helicosporium sexuale*	MFLUCC 16-1244^T^	MZ538503	MZ538537	MZ567082	MZ567111
*Helicosporium* sp.	NBRC 9014	AY916489	AY856903	N/A	N/A
*Helicosporium* sp.	Z17	PX220122	PX220124	N/A	N/A
*Helicosporium thailandense*	MFLUCC 18-1407^T^	MT627698	MN913718	MT954371	N/A
***Helicosporium tongrenense***	**GZCC 23-0026^T^**	**PQ098476**	**PQ098513**	**PX102603**	**PX102597**
***Helicosporium tongrenense***	**GZCC 25-0640**	**PX111180**	**PX111187**	**PX102604**	**PX102598**
*Helicosporium vegetum*	GZCC 23-0060	PP626584	PP639439	PP596342	PP596469
*Helicosporium vesicarium*	MFLUCC 17-1795^T^	MH558739	MH558864	MH550930	MH551055
*Helicosporium viridiflavum*	MFLUCC 17-2336^T^	MH558738	N/A	MH550929	MH551054
*Helicosporium viridisporum*	GZCC 22-2008^T^	OP508736	OP508776	OP698087	OP698076
*Helicotubeufia hydei*	MFLUCC 17-1980^T^	MH290021	MH290026	MH290031	MH290036
*Helicotubeufia jonesii*	MFLUCC 17-0043^T^	MH290020	MH290025	MH290030	MH290035
*Muripulchra aquatica*	MFLUCC 15-0249^T^	KY320532	KY320549	N/A	N/A
*Neoacanthostigma fusiforme*	MFLUCC 11-0510^T^	KF301529	KF301537	N/A	N/A
*Neochlamydotubeufia fusiformis*	MFLUCC 16-0016^T^	MH558740	MH558865	MH550931	MH551059
*Neohelicomyces acropleurogenus*	CGMCC 3.25549^T^	PP626594	PP639450	PP596351	PP596478
*Neohelicomyces aquaticus*	MFLUCC 16-0993^T^	KY320528	KY320545	KY320561	MH551066
*Neohelicosporium acrogenisporum*	MFLUCC 17-2019^T^	MH558746	MH558871	MH550937	MH551069
*Neohelicosporium aquaticum*	MFLUCC 17-1519^T^	MF467916	MF467929	MF535242	MF535272
*Neomanoharachariella xizangensis*	KUNCC 23-15799^T^	OR803724	OR803722	OR813978	OR813975
*Parahelicomyces quercus*	MFUCC 17-0895^T^	MK347720	MK347934	MK360077	MK434906
*Parahelicomyces talbotii*	MFLUCC 17-2021^T^	MH558765	MH558890	MH550957	MH551091
*Tubeufia guttulata*	GZCC 23-0404^T^	OR030841	OR030834	OR046678	OR046684
*Tubeufia hainanensis*	GZCC 22-2015^T^	OR030842	OR030835	OR046679	OR046685

Note: Newly generated sequences are in bold. The superscript “^T^” indicates ex-type strains. “N/A” indicates unavailable data in GenBank.

Maximum likelihood (ML) analysis was conducted using the IQ-TREE web server (http://iqtree.cibiv.univie.ac.at/) with the best-fit substitution model automatically selected based on the Bayesian Information Criterion (BIC) (Nguyen et al. 2015). Bayesian inference (BI) analysis was performed using MrBayes on XSEDE (v.3.2.7a) via CIPRES (Stamatakis 2014). The aligned FASTA file was converted to NEXUS format using AliView (Daniel et al. 2010). The optimal substitution model for each dataset was selected with MrModeltest v.2.3 (Nylander et al. 2008). Posterior probabilities (BYPP) were determined based on Bayesian Markov chain Monte Carlo (BMCMC) sampling (Huelsenbeck and Ronquist 2001). Four simultaneous Markov chains were run for 10,000,000 generations, and trees were sampled every 1,000^th^ generation. The burn-in phase was set at 25%, and the remaining trees were used for calculating posterior probabilities (BYPP).

Phylogenetic trees were visualized using FigTree v.1.4.4 and subsequently edited with Adobe Illustrator CC 2019 (v.23.1.0; Adobe Systems, USA).

## ﻿Phylogenetic results

**Figure 1.**
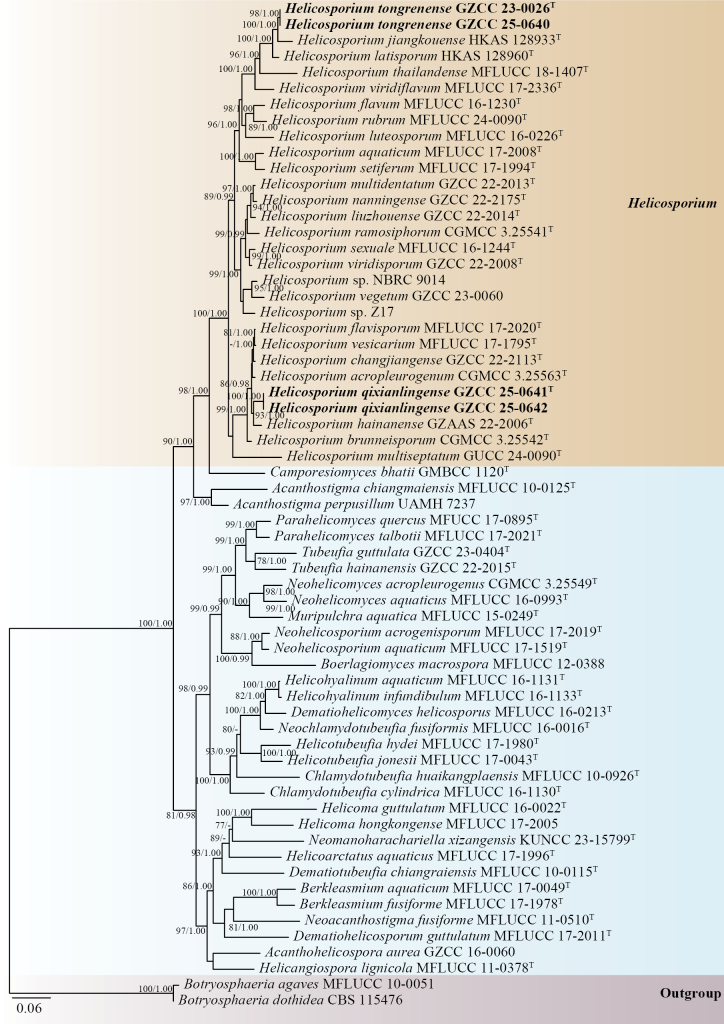
Phylogenetic tree generated using RAxML analysis based on the concatenated ITS, LSU, *tef*1-α, and *rpb*2 sequence data. Bootstrap support values (MLBS) ≥ 75% and Bayesian posterior probabilities (BYPP) ≥ 0.95 are indicated near the nodes as MLBS/BYPP, respectively. A hyphen (–) denotes support values below 75% for ML and posterior probabilities below 0.95 for BI. *Botryosphaeria agaves* (MFLUCC 10–0051) and *B. dothidea* (CBS 115476) were used as outgroups. Ex-type strains are marked with “^T^,” and newly obtained isolates are highlighted in bold black font.

The phylogenetic positions of the four novel strains were assessed using a multilocus phylogenetic approach. The concatenated sequence matrix comprised 3,346 nucleotide positions (ITS: 1–539, LSU: 540–1389, *tef*1-α: 1390–2301, and *rpb*2: 2302–3346), incorporating 61 ingroup taxa and two outgroup taxa, *Botryosphaeria agaves* (MFLUCC 10–0051) and *B. dothidea* (CBS 115476). Both maximum likelihood (ML) and Bayesian inference (BI) analyses of the combined ITS, LSU, *tef*1-α, and *rpb*2 datasets yielded congruent tree topologies. The best-scoring ML tree (Fig. 1) exhibited a final log-likelihood value of –34,578.071.

Phylogenetic analyses of the phylogram (Fig. 1) revealed that our collections include two new species of *Helicosporium* within the Tubeufiaceae (Tubeufiales, Dothideomycetes). Isolates GZCC 25–0641 and GZCC 25–0642 formed a sister clade to *Helicosporium hainanense* (GZAAS 22–2006), with robust support of 93% MLBS and 1.00 BYPP. Furthermore, GZCC 23–0026 and GZCC 25–0640 clustered together, and this clade was sister to *H. jiangkouense* (HKAS 128933), with 100% MLBS and 1.00 BYPP support.

### ﻿Taxonomy

#### ﻿ Helicosporium qixianlingense

Taxon classificationFungiTubeufialesTubeufiaceae

S. Bai, L.J. Chen, & J. Ma sp. nov.

95DDAD3A-5300-59F8-A508-C0298E766624

904205

Fig. 2

##### Etymology.

‘‘*qixianlingense*” refers to the place ‘‘Qixianling Hot Spring National Forest Park,” from where the holotype was collected.

##### Holotype.

GZAAS 25–0671

##### Description.

***Saprobic*** on decaying wood in a terrestrial habitat. **Sexual morph** Undetermined. **Asexual morph** Hyphomycetous, helicosporous. ***Colonies*** on natural substrate superficial, effuse, gregarious, yellowish green to brown. ***Mycelium*** partly immersed, partly superficial, composed of pale brown to brown, branched, septate, guttulate, smooth hyphae. ***Conidiophores*** 132–144 × 3.7–4.8 μm (x̄ = 139 × 5.5 μm, n = 30), macronematous, mononematous, erect, cylindrical, long, straight or slightly flexuous, simple, septate, brown to dark brown at base, paler towards the apex, smooth, thick-walled. ***Conidiogenous cells*** 4.5–10 × 3–4.4 μm (x̄ = 8 × 3.6 μm, n = 30), holoblastic, monoblastic, or polyblastic, integrated, intercalary, cylindrical, denticulate, arising laterally from the lower portion of conidiophores as tiny bladder-like protrusions (3–4 µm long, 2–2.7 µm wide), with each bearing 1–2 tiny sporogenous conidiogenous loci, hyaline to pale brown, smooth. ***Conidia*** solitary, pleurogenous, helicoid, tapering towards the rounded ends, developing on bladder-like protrusion, 11.7–12 μm in diameter, and conidial filament 1.2–2.1 μm wide (x̄ = 11.9 × 1.7 μm, n = 30), 72.5–84 μm long (x̄ = 76.5 μm, n = 35), up to 4^1^/_2_ times, becoming loosely coiled when the conidia are young and not becoming loose when mature in water, indistinctly multi-septate, guttules, hyaline, smooth-walled.

**Figure 2.**
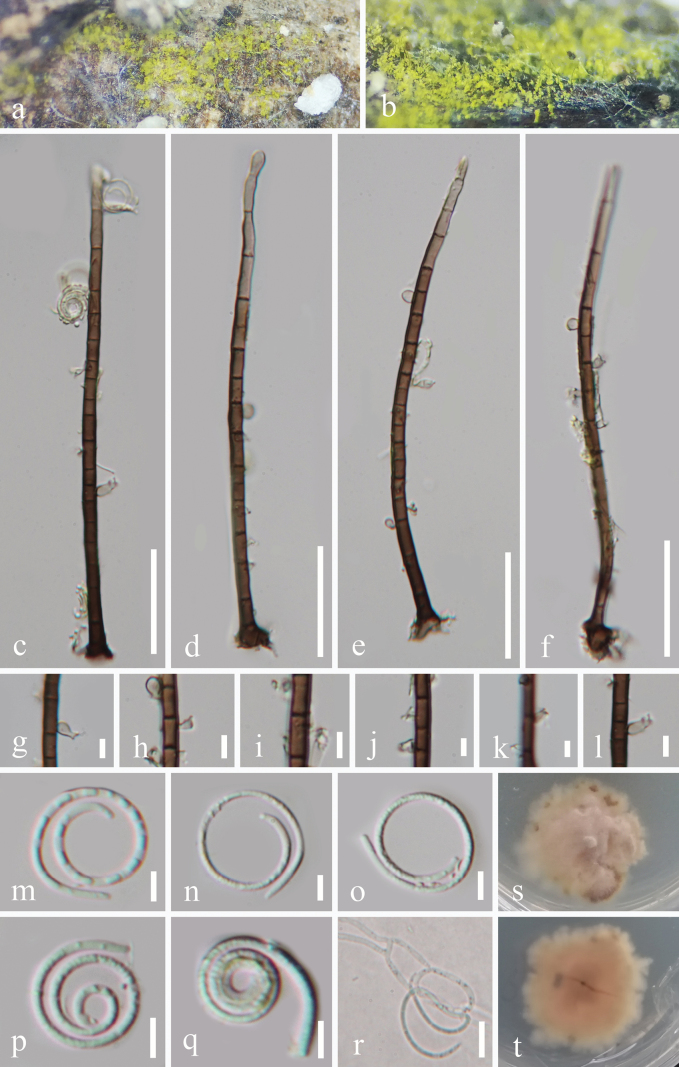
*Helicosporium qixianlingense* (GZAAS 25–0671, holotype). a, b. Colonies on the host surface; c–f. Conidiophores and conidiogenous cells; g–l. Conidiogenous cells; m–q. Conidia; r. A germinated conidium; s, t. Colonies on PDA from above and below after 44 days of incubation at room temperature. Scale bars: 30 μm (c–f); 10 μm (r); 5 μm (g–q).

##### Culture characteristics.

Conidia germinating on PDA within 11 hours, producing germ tubes from the conidial body. Colony on PDA reaching 3.2 cm in diameter after 44 days at room temperature (approximately 25 °C), circular or irregular, umbonate, with an undulate margin, and pale brown to light pinkish in color.

##### Material examined.

• China, Hainan Province, Baoting Li and Miao Autonomous County, Qixianling Hot Spring National Forest Park, on decaying wood in a terrestrial habitat, 2 November 2024, Jian Ma Q23 (GZAAS 25–0671, holotype), ex-type living cultures GZCC 25–0641; *Ibid*., Q39 (GZAAS 25–0672, paratype), living culture GZCC 25–0642.

##### Notes.

In the phylogenetic analyses (Fig. 1), our isolates (GZCC 25–0641 and GZCC 25–0642) formed a sister clade to *Helicosporium hainanense* (GZAAS 22–2006) with 93% MLBS and 1.00 BYPP support. However, nucleotide polymorphisms in the ITS, LSU, *tef*1-α, and *rpb*2 sequence data between GZCC 25–0641 and *H. hainanense* (GZCC 22–2006) revealed nucleotide differences of 11/432 bp (2.5%, with no gaps), 5/814 bp (0.6%, with no gaps), 11/906 bp (1.2%, with no gaps), and 39/1,045 bp (3.7%, with no gaps), respectively. Furthermore, *Helicosporium qixianlingense* (GZAAS 25–0671) differs from *H. hainanense* (GZAAS 22–2006, ex-type) by its longer conidia (72.5–84 μm *vs.* 55–60 μm), narrower conidial filament (1.2–2.1 μm *vs.* 2–3 μm), and more coiled times (up to 4^1^/_2_ times *vs.* 2^1^/_4_–2^3^/_4_ μm times) (Lu et al. 2022a). Therefore, based on both molecular and morphological evidence, we introduce *Helicosporium qixianlingense* as a novel species.

#### ﻿ Helicosporium tongrenense

Taxon classificationFungiTubeufialesTubeufiaceae

S. Bai, L.J. Chen, & J. Ma sp. nov.

77504171-AE71-5F29-BC9D-47EDBB4A5ED0

904206

Fig. 3

##### Etymology.

‘‘*tongrenense*” refers to the place ‘‘Tongren City,” from where the holotype was collected.

##### Holotype.

HKAS 128925

##### Description.

***Saprobic*** on decaying wood in a terrestrial habitat. **Asexual morph** Undetermined. **Sexual morph**: ***Ascomata*** superficial, seated on a subiculum, solitary, scattered, globose to subglobose, bright reddish yellow to brown yellow, with central narrow ostiole, no observed setae. ***Peridium*** composed of several layers of brown to dark brown cells of textura angularis, outer layer brown cells, and inner layer pale brown to hyaline cells. ***Hamathecium*** comprising numerous, filiform, septate, branched, hyaline pseudoparaphyses. ***Asci*** 90.5–143 × 11.5–14.5 µm (x̄ = 107.5 × 13 μm, n = 20), 8-spored, bitunicate, fissitunicate, cylindrical to clavate, short-pedicellate, apically rounded, basally flexious. ***Ascospores*** 37–48 × 3.5–5.5 µm (x̄ = 43 × 4 μm, n = 20), overlapping 2–3 seriate, elongate-fusiform, tapering towards narrow, widest at the central part, subacute ends, straight to slightly curved, guttules, multi-septate, hyaline, smooth-walled.

**Figure 3.**
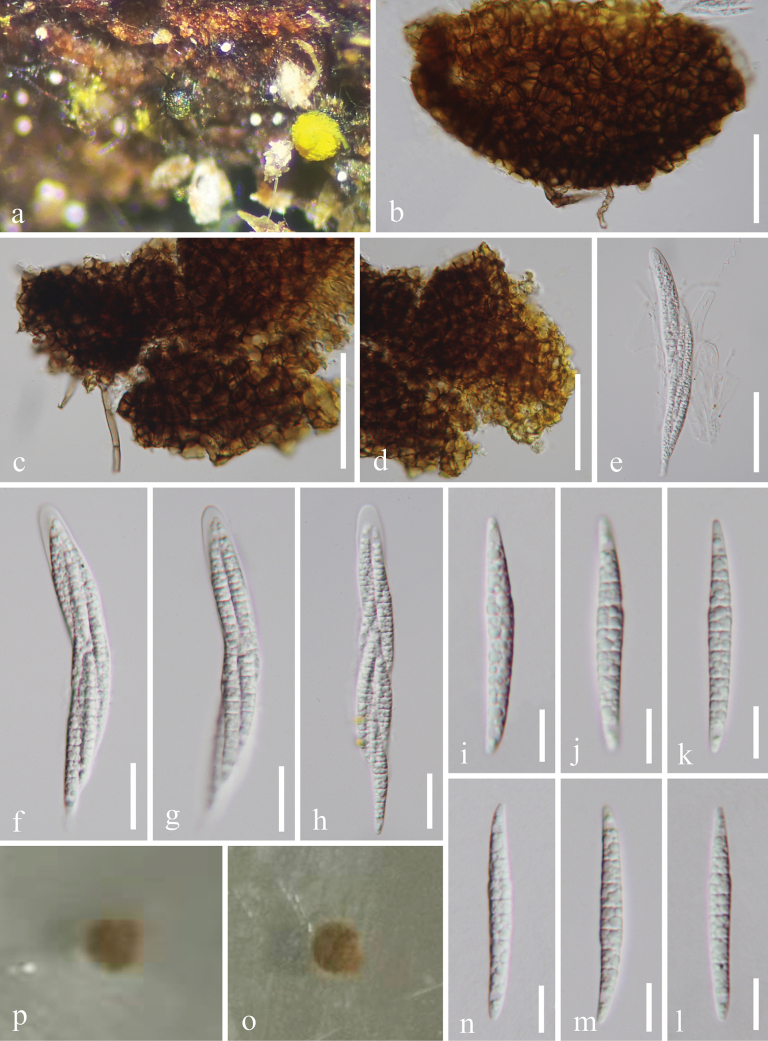
*Helicosporium tongrenense* (HKAS 128925, holotype). a. Ascomata on host surface; b–d. Vertical sections of ascomata and peridium; e–h. Asci; i–n. Ascospores; o, p. Colonies on PDA from above and below after 3 months of incubation at room temperature. Scale bars: 50 µm (b–e), 20 µm (f–h), 10 µm (i–n).

##### Culture characteristics.

Ascospores germinating on PDA, producing germ tubes within 19 hours. Colony on PDA reaching 0.7 cm diameter after 90 days at room temperature (approximately 25 °C), circular or irregular, flat, with an entire margin, and pale brown to yellowish brown in color.

##### Material examined.

• China, Guizhou Province, Tongren City, Jiangkou County, on decaying wood in a terrestrial habitat, 20 May 2022, Xia Tang, JK1.1 (HKAS 128925 = GZAAS 23–0027, holotype), ex-type living cultures GZCC 23–0026; *Ibid*., JK4 (GZAAS 25–0670, paratype), living culture GZCC 25–0640.

##### Notes.

Morphologically, *Helicosporium tongrenense* (HKAS 128925) resembles *H. flavum* (MFLU 17–0704) in having solitary, scattered, globose to subglobose, bright reddish-yellow to brown-yellow ascomata; 8-spored, bitunicate, fissitunicate, cylindrical to clavate asci; and elongate-fusiform, straight to slightly curved, multi-septate, hyaline ascospores (Brahmanage et al. 2017). However, *H. tongrenense* (HKAS 128925) differs from *H. flavum* (MFLU 17–0704) by shorter ascospores (37–48 μm *vs.* up to 60 μm) (Brahmanage et al. 2017). Additionally, *H. flavum* possesses short, setae-like projections, which are absent in *H. tongrenense* (Brahmanage et al. 2017). Phylogenetically, our isolates (GZCC 23–0026 and GZCC 25–0640) form a sister clade to *H. jiangkouense* (HKAS 128933), with 100% MLBS and 1.00 BYPP support. However, comparison of the ITS, LSU, *tef*1-α, and *rpb*2 sequence data between *H. tongrenense* (GZCC 23–0026) and *H. jiangkouense* (HKAS 128933) revealed nucleotide base differences of 26/526 bp (4.9%, including 1 gap), 51/834 bp (6.1%, including 3 gaps), 1/884 bp (0.1%, including 1 gap), and 1/916 bp (0.1%, with no gaps), respectively. Notably, the asexual morph of *H. jiangkouense* is the only form observed to date (Ma et al. 2024b). Therefore, based on both multigene phylogenetic analyses and morphological differences, we introduce *Helicosporium tongrenense* as a novel species.

## ﻿Discussion

Based on molecular data and/or morphological characteristics, the genus *Helicosporium* currently comprises 32 species, including our new species, *H. qixianlingense* and *H. tongrenense* (Hsieh et al. 2021; Xiao et al. 2023; Ma et al. 2024b; Peng et al. 2025; Sun et al. 2025). Among the known *Helicosporium* species, 26 are reported exclusively from the asexual morph, three (*H. multiseptatum*, *H. rubrum*, and *H. tongrenense*) solely from the sexual morph, and three (*H. flavum*, *H. sexuale*, and *H. vegetum*) have been documented in both sexual and asexual morphs (Nees 1817; Linder 1929; Samuels and Muller 1979; Barr 1980; Goos 1989; Morgan-Jones and Goos 1992; Spatafora et al. 2006; Tsui et al. 2006; Zhao et al. 2007; Boonmee et al. 2014, 2021; Lu et al. 2017a, 2018b, 2022a; Dong et al. 2020; Hsieh et al. 2021; Xiao et al. 2023; Ma et al. 2024b; Peng et al. 2025; Sun et al. 2025). Seven species—*Helicosporium albidum*, *H. casuarinae*, *H. decumbens*, *H. favidum*, *H. melghatianum*, *H. murinum*, and *H. neesii*—are currently known only from morphological descriptions but lack molecular data (Grove 1886; Linder 1929; Moore 1957; Matsushima 1985; Goos 1989; Zhao et al. 2007; Dharkar et al. 2010; Lu et al. 2018b; Hsieh et al. 2021; Ma et al. 2024b).

Previous studies have reported that secondary metabolites of *Helicosporium* species exhibit antibacterial activity against both bacteria and fungi (Kim et al. 2003; Lee et al. 2006, 2013; Choi et al. 2012). Our strains, GZCC 23–0026 and GZCC 25–0640, were successfully isolated using the single-spore isolation method of Lu et al. (2022b); however, the colonies exhibited slow growth, reaching only 0.7 cm in diameter after 90 days of incubation. Similarly, Ma et al. (2024b) employed the same method for two newly described asexual *Helicosporium* species, but the cultures yielded only sufficient material for DNA extraction. The alternative method of crushing the substrate and incorporating it into PDA medium to mimic natural growth conditions has proven useful for cultivating helicosporous fungi lacking available strains, as well as species that are difficult to grow on artificial media (Lu et al. 2022b; Ma et al. 2024b). Nonetheless, further optimization of culture media is necessary to accelerate mycelial growth in helicosporous fungi—thereby improving the acquisition of viable strain resources.

The asexual morphs of *Helicosporium* exhibit considerable variation in conidial filament width (Linder 1929; Boonmee et al. 2014, 2021; Lu et al. 2017a, 2018b, 2022a; Dong et al. 2020; Hsieh et al. 2021; Xiao et al. 2023; Ma et al. 2024b). For example, the conidial filaments of *H. setiferum* (GZAAS 23–0154) measure 1.5–2.5 μm in width, those of *H. flavum* (GZAAS 23–0491) are 3–4 μm wide, and those of *H. jiangkouense* (HKAS 128933) range from 4.5–6.5 μm (Ma et al. 2024b). Moreover, we observed that conidia with wider filaments maintain a tightly coiled structure in water, whereas those with narrower filaments tend to be loosely coiled or uncoiled (Linder 1929; Boonmee et al. 2014, 2021; Lu et al. 2017a, c, 2018b, 2022a; Dong et al. 2020; Hsieh et al. 2021; Xiao et al. 2023; Ma et al. 2024b). These observations suggest that the width of the conidial filaments is a key factor influencing whether *Helicosporium* conidia remain tightly coiled, become loosely coiled, or uncoil in water.

## Supplementary Material

XML Treatment for Helicosporium qixianlingense

XML Treatment for Helicosporium tongrenense

## ﻿Additional information

### Use of AI

No use of AI was reported.

### Author ORCIDs

Song Bai https://orcid.org/0000-0002-1972-2834

Fang Wang https://orcid.org/0000-0002-3341-6788

Su-Ran Wan https://orcid.org/0009-0000-1164-6921

Xiao-Kang Lv https://orcid.org/0009-0006-7254-8747

Li-Jun Chen https://orcid.org/0009-0004-8562-875X

Rong Wu https://orcid.org/0000-0002-4946-8806

Jian Ma https://orcid.org/0009-0008-1291-640X

### Data availability

All of the data that support the findings of this study are available in the main text.
